# Probiotic Fermentation of Defatted Cottonseed Meal for Sustainable Foods and Non-Food Applications

**DOI:** 10.3390/microorganisms13051020

**Published:** 2025-04-29

**Authors:** Zhanqiang Yan, Tian Li, Gen Zou, Xiaoling Zhang, Lingbo Qu, Yongjun Wei

**Affiliations:** 1 School of Medicine, Huanghe Science & Technology University, School of Chemical Engineering, School of Pharmaceutical Sciences, Zhengzhou University, Zhengzhou 450001, China; 2 Zhengzhou Research Base, State Key Laboratory of Cotton Bio-Breeding and Integrated Utilization, Laboratory of Synthetic Biology, Zhengzhou University, Zhengzhou 450001, China; 3 Institute of Edible Fungi, Shanghai Academy of Agricultural Sciences, Southern Key Laboratory of Edible Fungus Resource Utilization, Ministry of Agriculture, National Engineering Research Center of Edible Fungi, Shanghai 201403, China; 4 Food Laboratory of Zhongyuan, Zhengzhou University, Zhengzhou 450001, China; 5 Center for Lipid Biosynthetic Engineering, Muyuan Laboratory, 110 Shangding Road, Zhengzhou 450016, China

**Keywords:** cottonseeds, plant protein, gossypol removal, probiotic fermentation, solid-state fermentation

## Abstract

Cottonseed is a valuable source of high-quality proteins and oils. Defatted cottonseed meal (DCSM), a by-product of cottonseed oil extraction, holds significant potential as a sustainable protein resource. This review outlines the chemical composition, structural features, and unique properties of cottonseed, with a focus on its inherent antinutritional factors, such as gossypol. Strategies for enhancing the utilization of DCSM as a protein source are systematically evaluated, including physical, chemical, and biological methods used to eliminate or reduce antinutritional components. Among these, microbial fermentation, particularly solid-state fermentation, is highlighted as a promising, eco-friendly approach for detoxification and nutritional improvement. This review further discusses critical factors influencing the removal of anti-nutritional compounds, such as pretreatment methods, fermentation parameters, and microbial strains. The efficacy of probiotic strains (e.g., *Bacillus* and yeasts) in enhancing the protein digestibility, amino acid profiles, and functional properties of DCSM is discussed. Additionally, recent advances in the application of fermented cottonseed protein in foods (e.g., animal feed, functional peptides, and food additives) and non-food sectors (e.g., biofuels and bioplastic) are explored. The integration of probiotic-driven fermentation processes is proposed as a strategy to exploit the full nutritional and economic potential of DCSM, paving the way for its broader and sustainable use in foods and non-food applications.

## 1. Introduction

Cotton (*Gossypium* spp.) is a globally cultivated crop with widespread agricultural significance [1]. According to the 2022 database of the Food and Agriculture Organization of the United Nations (FAO), cotton was grown on 31.4 million hectares worldwide, yielding 69.7 million tons of production. As a primary source of natural fiber for the textile industry, cotton is cultivated in over 75 countries. In particular, China, India, and the United States collectively account for more than 70% of global cotton production [2]. The cotton plant comprises several components, including cotton fiber, cottonseed, cotton hull, cottonseed shell, and cotton stalk [3]. While cotton is primarily grown for its fiber, approximately 150 kg of cottonseed is produced for every 100 kg of lint fiber obtained during ginning [4]. Cottonseed is rich in lipids, proteins, carbohydrates, and minerals [5]. It contains 17–22% oil, which is predominantly utilized in the food industry. The extraction of oil yields defatted cottonseed meal (DCSM) as a valuable by-product [6].

DCSM is recognized as one of the most promising sources of plant protein. DCSM typically contains 20–70% protein by dry weight, with the exact content varying significantly depending on the preparation and analytical methods used. In some cases, cottonseed protein can achieve nearly 100% [4]. Due to its high protein content, low cost, abundant availability, and ease of processing, DCSM is widely utilized in feeds for domestic animals and fish [7,8]. However, cottonseed protein contributes about 5% to the global protein supply. This limited utilization is primarily attributed to several challenges associated with DCSM, including poor palatability, low digestibility, and an unappealing dark color. Additionally, DCSM contains some anti-nutritional factors, such as gossypol, cyclopropenoid fatty acids, phytic acid, and tannins [9,10,11]. Among these, gossypol presence in feed negatively impacts animal growth, development, and reproductive health, thereby restricting the broader use of cottonseed in animal nutrition [12,13].

The growing demand for soybean meal and animal-based proteins has positioned DCSM as a competitive and sustainable alternative protein source for animal feed. Cottonseed protein isolates have diverse applications, including protein supplements [14], bio-based adhesives [15,16,17], and bioplastics [18,19]. Additionally, cottonseed shells and cotton stalks can serve not only as dietary cellulose supplements in animal feed but also as raw materials for producing a range of value-added products, including bio-based materials, chemicals, and biofuels [20,21].

**Figure 1:**
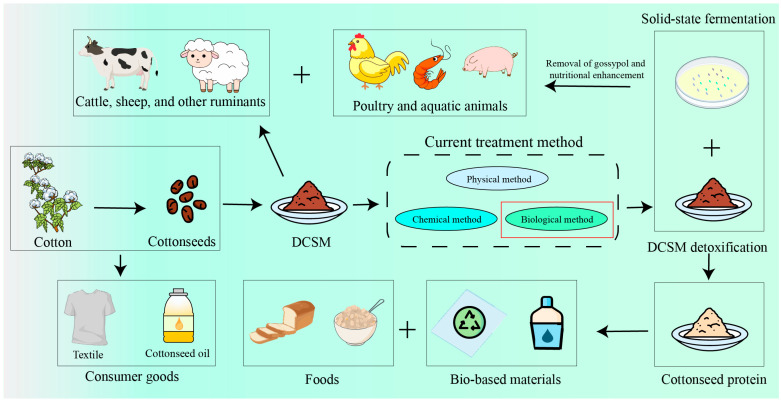
Cotton and its by-products demonstrate remarkable versatility across multiple industries. Cotton is primarily utilized in the textile industry, while cottonseed is commonly processed for oil extraction. DCSM is widely used as animal feed. Large ruminants can tolerate gossypol without pretreatment. The removal of gossypol from DCSM through biological methods (solid-state fermentation) has significantly enhanced its utilization as feed for both small ruminants and non-ruminants. Furthermore, cottonseed protein extracted from detoxified DCSM has been explored as a valuable supplement for various foods and biomaterials.

In this review, the chemical composition and biological characteristics of various parts of the cotton plant are outlined, and the application of cottonseed protein in animal feed is introduced. Furthermore, this study systematically investigated the applications of DCSM in pharmaceutical, industrial, and foods (Figure 1). The technological advancements and commercialization potential of cotton by-product processing strategies are analyzed, aiming to establish a theoretical foundation for innovative utilization strategies that enhance the value chain integration of DCSM and optimize its economic benefits.

## 2. Chemical Composition, Structure, and Characteristics of Cottonseed

**Figure 2:**
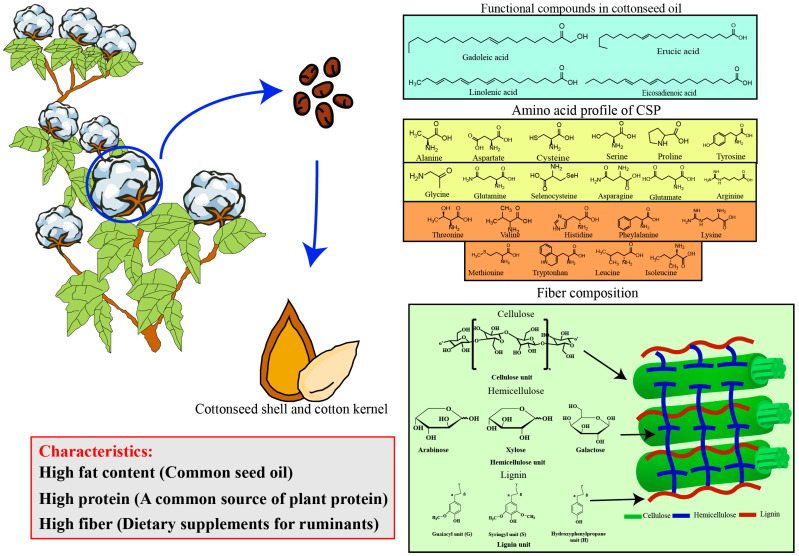
Specific properties and chemical composition of cottonseed. Cottonseeds consist of approximately 18% oil and 22–24% protein. Cottonseed oil is rich in functional fatty acids, as well as diverse amino acids. Additionally, cottonseed shells are abundant in cellulose, hemicellulose, and lignin, making them a valuable source of renewable lignocellulosic biomass.

Cottonseeds are primarily composed of protein, fat, and fiber (Figure 2). Among oilseed crops, cottonseed ranks as the fifth-largest source of protein following soybean, canola, palm, and sunflower [22,23]. In addition to fiber, cottonseed yields hulls (22%), liters (7%), and DCSM (50%) [5,24]. Due to the different technologies of oil extraction, the obtained components have certain differences.

### 2.1. DCSM Obtained After Cottonseed Oil Extraction

DCSM is rich in protein, fiber, and other nutrients (Figure 2). It is often used in food and feed production. However, nutrient concentrations in different samples vary [25]. These differences may be due to different agronomic practices (such as fertilization), different degrees of cortex removal, the ratio of the skin to the shell, and processing methods during oil extraction [26,27]. DCSM was added to enhance soybean meal utilization by 50% in lactating dairy cow diets [28].

Lysine is the first limiting amino acid in cotton protein (Figure 2). During oil extraction processing, gossypol forms stable complexes with the lysine amino group, thereby reducing lysine bioavailability and resulting in diminished lysine digestibility in cottonseed-derived products [29]. The content of gossypol had a negative effect on the amino acid digestibility of DCSM [30]. When the gossypol content was 1.52%, the true digestibility coefficient of essential amino acids in DCSM was 73.9% to 91.8% [11]. Higher dietary inclusion of DCSM increased the concentration of free gossypol, changed the dietary amino acid composition, and limited the growth of goslings aged 1–20 days [31]. Therefore, reducing the level of gossypol in cottonseed can increase feed yield.

### 2.2. Antinutritional Factors—Gossypol

Gossypol, a phenolic terpenoid aldehyde biosynthesized in pigment glands, exhibits dual antioxidant and phytotoxic properties. This specialized metabolite functions as an endogenous defense compound against biotic stressors (e.g., phytophagous insects, microbial pathogens) while concurrently enhancing plant resilience to abiotic environmental challenges through adaptive biochemical coordination [32,33]. Cottonseed generally contains 0.02–6.64% gossypol based on dry weight depending on the cotton variety and the region and climate [5,34] and is synthesized by the pigment glands of cotton plants in the mallow family [35]. Gossypol exhibits anti-physiological activity, including impairing somatic growth, disrupting developmental homeostasis, and suppressing reproductive efficiency in animal species.

Gossypol is available as bound gossypol and free gossypol in cotton. Free gossypol has active aldehyde and hydroxyl groups and can harm animal blood vessels, cells, and nerves, cause animal physiological function disorders, and reduce the reproductive ability of male animals. Bound gossypol is bound to proteins, amino acids, or other substances. Therefore, bound gossypol is not easily absorbed by animals and is less toxic to animals [34,36]. Three principal mitigation strategies are routinely implemented during DCSM valorization for animal nutrition, including mechanical processing, chemical treatment, and microbial fermentation [37]. The limit of free gossypol in DCSM protein products that are safe for use in non-ruminants is 450 ppm, which is set by the United States Food and Drug Administration (US FDA) and the World Health Organization (WHO) [5].

### 2.3. Green and Sustainable Plant Protein–Cottonseed Protein

Decorticated cottonseed meal containing concentrated protein content exceeding 44% represents a high-quality plant-derived protein matrix for industrial applications. The cottonseed protein contains high salt-soluble protein, followed by water-soluble protein and alkali-soluble protein [5,38]. The surface properties of proteins are based on different functional properties, such as wettability, dispersion, oxidative stability, fluidity, and rehydration [39]. Arginine accounts for 15–34% of total cottonseed protein, and another 8 essential amino acids account for approximately 5% of total protein. Among the non-essential amino acids, glutamic is the highest, accounting for 10% of total cottonseed protein. Quantitative amino acid profiling demonstrated that histidine, isoleucine, leucine, lysine, methionine, phenylalanine, threonine, and valine constituted 30.0% and 28.1% of the total amino acid content in alkali-soluble and salt-soluble protein fractions, respectively [40]. The essential amino acids in glandless DCSM were 26%, which is higher than that in soy protein (17%), showing that DCSM might be an excellent source of protein for animal consumption.

Dietary fiber is defined as non-digestible carbohydrate with lignin, which includes non-digestible starch polysaccharides, hydrocolloids, resistant starch, resistant oligosaccharides, and lignin related to dietary fiber polysaccharides [41]. Cellulose, hemicellulose, and lignin are the main sources of cotton dietary fiber (Figure 2) [42]. Dietary fiber is one of the main components of agricultural by-products, which can be divided into soluble dietary fiber and insoluble dietary fiber [43]. Dietary fiber can affect the structure of animal intestinal microbiota [44,45]. Thus, understanding the physiological function of dietary fiber in animal nutrition and the composition of its metabolites helps promote the development of animal feed.

## 3. Approaches for Efficient Utilization of DCSM

**Figure 3:**
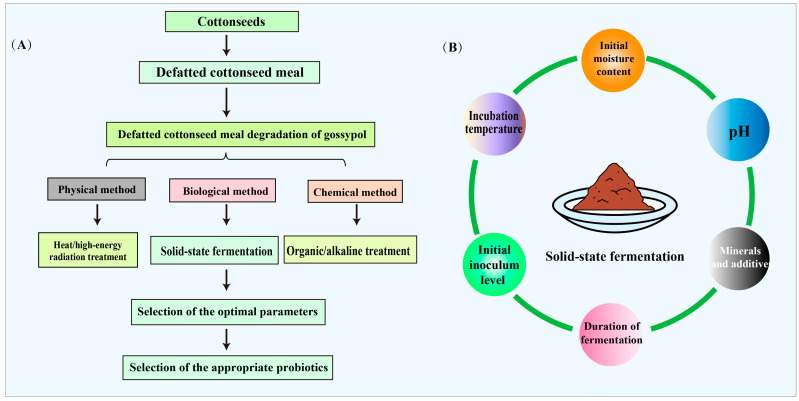
The DCSM detoxification methods and the influencing factors of solid-state fermentation. (**A**), the detoxification methods of DCSM include physical, chemical, and biological methods. Solid-state fermentation is a typical way to ferment DCSM. (**B**), solid-state fermentation is affected by the initial moisture content, pH, incubation temperature, the initial inoculum level, the duration of fermentation, and minerals and additives.

DCSM is a by-product of the cotton fiber industry, and its use in animal feed is limited due to the toxicity of gossypol. The sensitivity of different animals to gossypol varies; The EU Directive 2002 L00032 limits free gossypol in cottonseed cake feed to 20 ppm for laying hens and piglets, 100 ppm for poultry and calves, and 500 ppm for cattle, goats, and sheep [46]. DCSM should be detoxified before being used in feed. The common pretreatment methods for gossypol in DCSM include physical, chemical, and biological methods (Figure 3).

### 3.1. Physical Methods for the Degossypolization of DCSM

The available physical methods for the removal of free gossypol include heat treatment, separation of cottonseed pigment glands, and high-energy radiation. Under thermal hydrolysis processing conditions, free gossypol undergoes covalent conjugation with amino acid residues or polypeptide chains, forming bound gossypol derivatives through chemisorption-mediated detoxification mechanisms [29]. This method could reduce the gossypol in DCSM by 91.1% [47]. However, this method reduces the protein content in the kernel and the fatty acid content in the oil, which limits its application in DCSM detoxification. Cottonseed with glands can be treated with physical methods to remove gossypol-bearing glands, such as the air classification process or liquid cyclone process [48]. Liquid cyclone process can produce edible DCSM with 0.04% or less free gossypol [37]. The air classification process is designed to reduce gossypol content in DCSM by removing pigment glands, which was developed as a means to improve the liquid cyclone process [37]. Recently, gamma, and electron irradiation has been proven to be effective in reducing anti-nutrient factors of various plant sources [49,50], and this treatment does not cause damage to nutrients or form undesired products [51].

### 3.2. Chemical Methods for the Degossypolization of DCSM

The organic solvent extraction method is based on similar phase dissolution. The most commonly used solvents are acetone, ethanol, n–butanol, methanol, and dichloromethane. Gossypol has a strong affinity and forms strong bonds with divalent cations, especially iron. The addition of iron sulfate can enable feed containing safe levels of gossypol of various domestic animals [52]. Adding the same amount of dehydrated ferrous sulfate to DCSM reduces the level of free gossypol to 0.0001%. The free gossypol content of DCSM was effectively reduced when calcium hydroxide was used for the alkali treatment of DCSM. The use of calcium hydroxide (2%) and pressure-cooking treatment reduced the free gossypol content of DCSM in poultry feed [53]. However, calcium hydroxide generally reduces vitamins and the detoxification efficiency of gossypol removal [54].

### 3.3. Biological Methods for the Degossypolization of DCSM

**Figure 4:**
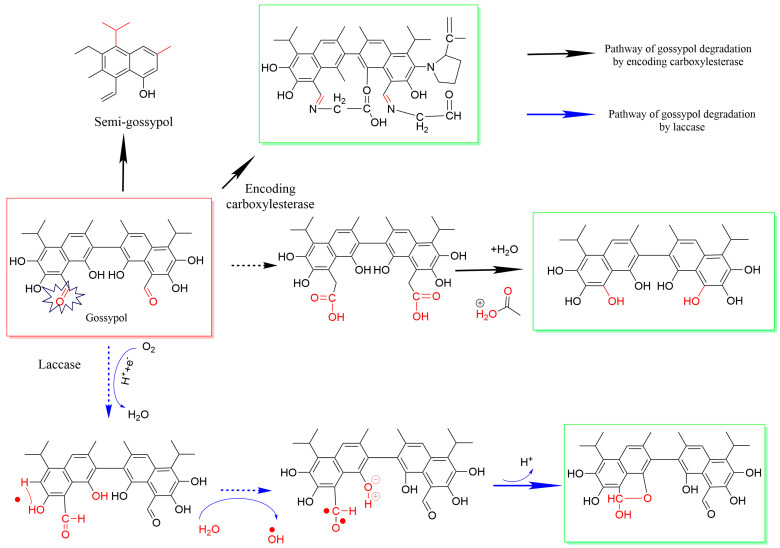
Enzymatic approaches to bind or eliminate the toxic aldehyde groups in gossypol. The dashed arrows indicate the inferred process of binding or removing the aldehyde groups responsible for gossypol toxicity, while the solid arrows represent the reported gossypol degradation steps.

Microbial fermentation could not only efficiently remove gossypol but also improve the nutritional values of cottonseed powder [55,56]. Biological methods include the genetic breeding method, the enzyme digestion method, and microbial fermentation. Gossypol and related terpenoids are produced and stored in the lysigenous glands in the cotton plant. Thus, the removal of “glandless mutant cotton” without lysigenous glands is a target for biological breeding. Though commercial breeding has been attempted, commercial glandless cotton varieties have failed due to the crop being more sensitive to pests than conventional cotton. Enzymatic hydrolysis is an effective method to improve the quality of cottonseed protein because the reaction conditions are non-irritating, have no impact on the nutritional value of amino acids, and produce low environmental pollution [57]. Enzymatic biocatalysts could bind or remove the toxic aldehyde group in gossypol (Figure 4) [58,59]. To elucidate the mechanism of the CarE CCE001a protein in the efficient degradation of gossypol, UPLC-QTOF/MS analysis was employed to detect gossypol degradation intermediates (Figure 4). The results suggest that binding to or removal of the toxic aldehyde group(s) in gossypol could effectively reduce its toxicity [58]. Laccase could help to degrade gossypol [59]. Laccase can catalyze the intramolecular cyclization of aldehyde and hydroxyl group of gossypols with o–semiquinone radical and produce free radical ·OH. Oxidation of the aldehyde group significantly reduced reproductive toxicity and hepatotoxicity [59]. The *Panus lecomtei* strain BRM044603 demonstrated a significant capacity to reduce free gossypol to 100 μg/g. Enzymatic and proteomic analyses indicated that the increase in laccase activity was correlated with the reduction of free gossypol and the degradation of gossypol to trace amounts [60]. Five engineered strains (GS115-LacA, GS115-LacB, GS115-LacC, KM711-LCC1, and GS115-Lcc2) were generated by expressing high-activity laccases. Among them, KM711-LCC1 exhibited the highest efficiency in gossypol degradation. The degradation performance of these laccases was positively correlated with temperature and pH [61].

Solid-state fermentation is a fermentation process that harnesses microorganisms under conditions of low moisture. Liquid fermentation is a fermentation process that involves large-scale microbial cultivation through a flow environment with proper dissolved gas and nutrients [62,63]. The starter, substrate, and fermentation conditions might change the nutritional properties of fermented feed [64]. Yeast species, such as *Saccharomyces cerevisiae*, are the most used microorganisms for DCSM fermentation [65,66]. *Bacillus* spp. exhibit free gossypol biodegradation capacity, and specific strains could enhance the nutritional quality of DCSM [56,67]. Certain lactic acid bacteria can reduce the content of anti-nutrient elements in DCSM and produce a large number of volatile substances, such as lactic acid and acetic acid, which can improve the palatability of feed. The secretion ability of lactic acid bacteria is weak, resulting in less improvement in the nutritional value of DCSM [68]. The insect gut is an important interface between the host and the external environment. It is possible to isolate and identify bacteria from the insect body which uses cotton leaves (including gossypol) as feed [69].

Microbial fermentation has emerged as a promising biotechnological approach for DCSM detoxification, functioning via dual mechanisms of biodegradation and molecular conjugation. The process requires scientific strain selection and targeted microbial treatment. Successful detoxification crucially depends on strain specificity (particularly gossypol-degrading enzyme production capacity) and optimized fermentation parameters including moisture content, temperature, and substrate pretreatment.

### 3.4. Factors Affecting the Degradation of Gossypol by Solid-State Fermentation

The initial moisture content, pH, incubation temperature, initial inoculum level, duration of fermentation, and mineral additives might affect the final anti-nutrient content of DCSM after solid-state fermentation (Figure 3). The moisture content of a fermentation substrate should be determined according to the properties of the substrate (particle size), microbial characteristics (anaerobic, aerobic, or facultative anaerobic), temperature, and time [64]. The initial moisture can affect the microbiota and flavor generation, and increasing moisture could enhance the *Lactobacillus* content and microbial stability during Baijiu production [70]. Elevateed moisture content inhibits the conversion efficiency of free gossypol to bound gossypol [71]. The metabolic activity of microorganisms is largely affected by the pH value of the medium [72].

Microorganisms have specific temperature ranges where they have optimal growth. Maintaining an optimal temperature is critical to shortening the stabilization phase of fermentation and maximizing product yield while minimizing by-product formation [73]. Furthermore, controlled thermal conditions enhance enzymatic reaction kinetics and promote microbial proliferation, thereby accelerating metabolic flux and substrate utilization efficiency [74]. A free gossypol degradation rate of 86.5% was achieved at 30 °C, while only 57% was achieved at 40 °C [5,75].

The initial inoculum level is another important factor affecting the biodegradation of free gossypol. A high concentration of *Candida tropicalis* degraded more than 80% free gossypol, while a low concentration degraded less than 60% free gossypol [5,75]. The duration of fermentation depends on characteristics such as the growth rate of the microorganism and the efficiency of free gossypol degradation. Shorter fermentation times may result in incomplete utilization of the substrate, thereby reducing the gossypol degradation rate, while fermentation beyond the optimal range may result in denaturation and subsequent deactivation of enzymes associated with free gossypol degradation due to interactions with other compounds/by-products formed during the process.

Mineral supplementation was found to improve free gossypol detoxification efficiency and protein content. Phosphate contributes to the buffering capacity of the medium and is a component of nucleic acids, phospholipids, and coenzymes. The supply of potassium or sodium ions changes the osmotic pressure. Potassium is a major cation in microbial cells, especially as a cofactor for enzymes such as hexokinase phosphate.

### 3.5. Impact of Different Start Cultures on the Nutrient Content of DCSM

The different starter cultures affect the gossypol removal efficiency of DCSM (Table 1). The degradation of gossypol by microorganisms may be due to the use of gossypol as a carbon source or the conversion of free gossypol to bound gossypol. *Bacillus* species have been applied in gossypol degradation [66,76,77].

*B. subtilis*, *B. coagulans*, *Lactobacillus agilis*, and other bacteria increased the crude protein content of DCSM and degraded free gossypol after fermentation. The protein content in DCSM reaches 7.63% post *B. subtilis* BJ–1 bioprocessing [79]. The rumen-derived strain *L. agile* WWK129 achieved 83% degradation of gossypol in DCSM within 5 days [78]. After 14 days of solid-state fermentation with *B. subtilis* M-15, the gossypol degradation rate in DCSM reached 93.46%, while the acid-soluble protein content significantly increased to 13.26% [67]. Eight microbial strains exhibiting gossypol-degrading capabilities were isolated from DCSM, and the isolated *Meyerozyma guilliermondii* WST–M1 reduced the total gossypol and free gossypol content by 31.97% and 74.70%, respectively [71]. The fermentation of soybean meal by *B. amyloliquefaciens*, improved crude protein content and acid soluble protein concentration. Moreover, the antigenic protein was degraded during fermentation [92]. Extracellular enzymes were produced by bacteria during fermentation. *Bacillus* spp. produces industrially valuable enzymes such as protease and xylanase are produced [93].

Yeast is often used in fermentation processes due to its ease of culture, fast growth rate, and inherently high protein content [94]. Yeast demonstrates gossypol reduction in DCSM and improve nutrition and is currently widely applied in feed additives [80,81,82]. *Geotrichum candidum* G07 exhibits potent gossypol degradation capacity, with a detoxification efficiency of 78.9% after 48 h incubation at 30 °C [83]. Fermentation coupled with enzymatic hydrolysis of DCSM improves protein utilization efficiency and expands the application potential of unconventional proteins [84]. To improve the overall quality of DCSM, short-term (4 days) and long-term (14 days) fermentation using yeast strains caused an increase in total essential amino acid content (M = 44%) and total non-essential amino acid content (16–18%). In addition, a 17% reduction in gossypol is achieved [95]. The yeast is a potential source for protein in food, and the application of yeasts in DCSM fermentation might satisfy the requirement for future protein demand [96]. Fungi, such as *Aspergillus niger* and *Pycnoporus sanguineus*, are often used to ferment DCSM. *Trichoderma* spp. has the ability to synthesize proteins [97]. The detoxification efficiency of free gossypol varied according to the type and proportion of the strains used in DCSM fermentation.

Mixed culture enhances fermentation efficacy by producing high-activity hydrolases, increasing crude protein content, reducing the crude fiber levels, and improving the nutritional quality of detoxified DCSM [85,86]. Fermentation of DCSM using *B. subtilis* ST-141 and *Saccharomycetes* N5 can effectively reduce gossypol content and enhance acid-soluble protein concentration [87,90]. DCSM fermented with *Candida tropicalis* and *S. cerevisiae* provides a simplified processing approach for the animal feed industry [88]. Mixed fermentation can take advantage of the synergistic effect among different strains. Solid-state fermentation effectively converts anti-nutritional factors such as gossypol in DCSM and glucosinolates in rapeseed meal, enhancing dietary nutritional bioavailability, and confers potential application value in animal husbandry [89].

### 3.6. Application of Microbiome Engineering in Gossypol Degradation

**Figure 5:**
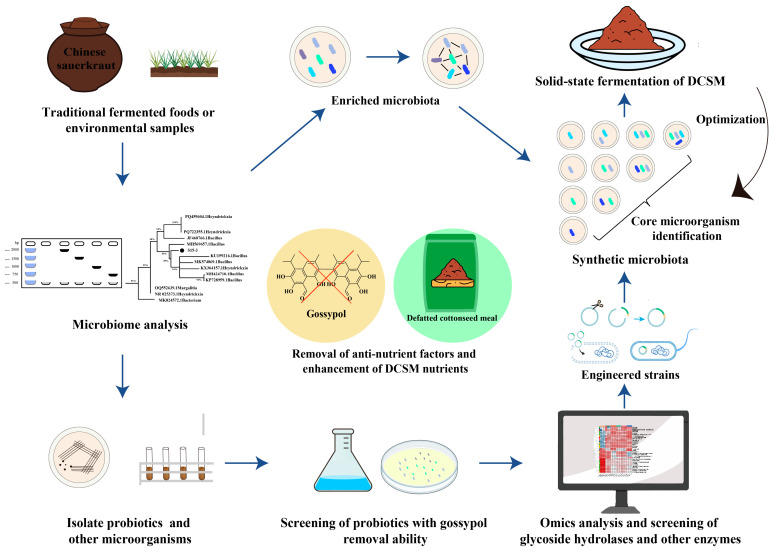
Screening and engineering gossypol degrading microorganisms. Microorganisms are first isolated and identified from fermented foods or environmental samples, followed by screening for strains capable of utilizing gossypol. Genomic and transcriptomic analyses are then employed to mine enzymes associated with gossypol metabolism, and genetic engineering is conducted to enhance metabolic efficiency. Subsequently, a synthetic microbiota was constructed through plate screening and applied for fermenting DCSM in industrial production. Besides, the natural microbiota with gossypol removal ability can be enriched with gossypol as substrate.

Although progress has been made in the probiotic-mediated fermentation of DCSM [6], the functional interplay between microbial consortia and gossypol within its structurally complex matrix remains largely uncharted. This knowledge gap necessitates the incorporation of multi-omics profiling and genetic engineering technologies [98], which hold the potential to elucidate the underlying mechanisms and thereby advance the integration use of DCSM. Gossypol-degrading microorganisms can be obtained through screening from fermented foods or natural environments, and engineered microorganisms via metabolic engineering approaches could be assembled into synthetic microbiota for testing and industrial application for DCSM (Figure 5). Through screening and selection of seven high-yielding protease-producing strains, microbial strains with superior activity in digesting free gossypol were identified [67].

Gene editing technology offers a novel alternative approach for bottom-up metabolic reprogramming of microbial systems [99,100]. The carboxylesterase (CarE)-encoding gene *cce001a* derived from *Helicoverpa armigera* was transformed into *Pichia pastoris* GS115. The target protein CarE CCE001a was successfully expressed, which exhibited effective degradation capability against free gossypol, achieving a degradation rate of 89% within 1.5 h [58]. Transcriptomic analysis of *C. tropicalis* ZD–3 revealed that the CTRG_04744 gene was significantly upregulated under gossypol stress. Subsequently, this gene was expressed in *P. pastoris*. The purified recombinant protein AKR_Z1 demonstrated efficient gossypol degradation capability, achieving a degradation rate of 92% within 48 h [101]. The development of gene editing and other advanced biotechnologies will enable microbes to acquire new capabilities, and these engineered microbes can significantly enhance fermentation effects [102,103]. Furthermore, integrating engineered probiotics with natural probiotics would generate a synthetic microbiota with high biomass transformation and gossypol removal ability [104,105].

## 4. Foods and Non-Food Applications of Cottonseed Protein

Cottonseed protein is produced through oil extraction, removal of gossypol, and other process stages. The cottonseed protein could reach up to 60% of DCSM. Cottonseed protein has great potential as a component of value-added industrial products and bioactive functional materials. Cottonseed protein isolate includes bioadhesives, food additives, enzyme preparations, bioplastics and films, antioxidant components/peptides, and antimicrobial peptides (Figure 1).

### 4.1. Cottonseed Protein as Food Source for Human Nutrition

The global annual production rate of cottonseed protein is about 10.8 million tons, which can meet the protein needs of the global population of 50 g of protein per day [106]. Nearly 23% of cotton seeds are made up of high-quality protein. The use of cottonseed protein in foods depends on gossypol content, nutritional value, and functional properties. The Food and Drug Administration (FDA) admits cottonseed proteins as food supplements if they have less than 0.8% bound gossypol or 0.045% free gossypol [107]. Therefore, reducing the gossypol content in cottonseed protein is a prerequisite for high-quality protein. The addition of DCSM increases the protein content of snacks. Extruded snacks prepared with 10% DCSM have 88% more protein and lower in fat [108]. The freeze-dried protein powder obtained under optimized conditions had higher crude protein content (93.1%), ultra-low gossypol, and no foodborne pathogens [109].

### 4.2. DCSM as Animal Protein Source

Low gossypol DCSM can be effectively used as feed for poultry and aquaculture. These animals can efficiently convert feed protein into edible animal protein. The transformation of ultra-low gossypol cottonseed protein into animal-derived proteins operates under defined protein conversion metrics, and the values exhibit species-specific variation [106]. The fermentation process reduced the content of gossypol and crude fiber and increased the content of certain beneficial lactic acid bacteria and crude protein in DCSM. The DCSM processed through fermentation could be used as an alternative protein source for poultry feed, which could reduce reliance on soy protein feed [110]. Converting cottonseed protein to animal protein reduced the risk of gossypol.

### 4.3. Non-Food Application of DCSM and Cottonseed Protein

The mechanical properties, water solubility, plasticizing properties, cross-linking behavior, and 3D structure of cottonseed protein affect the non-food applications of DCSM. These applications include adhesives, packaging, bioplastics, hydrogels, interface materials, and emulsification [4].

#### 4.3.1. Cottonseed Protein Used as an Adhesive

Certain adhesives on the market are urea–formaldehyde resins, which are derived from petroleum. Formaldehyde could cause serious health problems. Consequently, developing sustainable, renewable adhesive systems to replace petroleum-derived formaldehyde is necessary [111]. Cottonseed protein has been demonstrated in adhesive engineering as a viable wood binder, with its combination incorporating phosphoric acid exhibiting superior binding performance compared to soy protein [112]. The phosphorus/calcium was used as a protein modifier to improve the adhesive’s bond strength and water resistance. The interaction between amino acid and phosphorus enhanced the crosslinking property and improved the bonding property of cottonseed protein-based wood adhesive [15].

#### 4.3.2. Cottonseed Protein as Packaging Material

Films and coatings are used in the packaging of agricultural products such as fruits and vegetables. In the packaging industry, biodegradable materials have become the preferred material to replace plastic packaging. Protein-based biodegradable polymers are considered second-generation bioplastics [113], for they provide better mechanical support, and better protection against gas leaks [114]. A blended film by casting cottonseed protein and polyvinyl alcohol (PVA) was prepared and modified with different plasticizers. The degree of plasticizers is the interaction between proteins and polyvinyl alcohol, and the interaction changes the secondary structure of cottonseed protein. These blended films could be promising plastics for food packaging and flower-growing applications [115].

#### 4.3.3. Cottonseed Protein as a Substrate for Industrial Production of Enzymes

The enzymes produced by microorganisms from agro-industrial waste are cost-effective [116]. The solid substrate provides nutrients for the growth of microorganisms [117]. DCSM can be used as a substrate for microbial growth. When DCSM: wheat bran was used as a substrate for growing *Botryosphaeria* spp. AM 01 and *Saccharicola* spp. EJC 04 under solid-state fermentation conditions, cellulase and xylanases activities were detected, which might be used for sugarcane bagasse treatment [118]. Cottonseed cake was used as a nitrogen source to produce a fibrinolytic protease by *B. cereus*, which increased 71% protease activity [119].

The advanced processing of fermented cottonseed meal will drive the resource utilization of agricultural waste, which holds significant implications for ensuring sustainable development in animal husbandry and alleviating global feed protein resources through technological innovation.

## 5. Conclusions

DCSM is a valuable protein source with broad application potential. However, the presence of gossypol limits its widespread utilization. Solid-state fermentation offers a green, eco-friendly, and sustainable solution for gossypol removal, and the strains are essential for the fermentation. Microbiome and synthetic biology are expected to be the key approaches for the comprehensive utilization of DCSM. Through detoxification and high-value utilization processes, DCSM has emerged as an eco-friendly alternative to conventional raw materials across multiple fields, including food processing, livestock feed production, biofuel generation, and bioplastics manufacturing, while simultaneously addressing economic viability and environmental sustainability. In conclusion, through appropriate fermentation and other biological methods, it is feasible to further promote the sustainable development of the cotton industry and exploit the value of cottonseed in the future.

## Figures and Tables

**Table 1:** The effects of free gossypol detoxification by microbes using solid-state fermentation.

Microorganisms	Optimum Conditions					Free Gossypol Removed (%)	Improvement in Protein Content (%)	Reference
	Initial Moisture Content (%)	pH	Temperature (°C)	Duration of Fermentation (h)	Initial Inoculum Level (cells/g)			
*Bacillus subtilis* GH38	50	6.5	39	72	10^7^	78.86	4.98	[76]
*Bacillus subtilis* BJ–1	50	–	30	48	1% (*v*/*w*)	59.47	7.63	[77]
*Bacillus coagulans* S17	50	–	40	52	2.8 × 10^9^	81.83	10.09	[66]
*Lactobacillus agilis* WWK129	50	–	39	120	5% (*v*/*w*)	80.0	7.12	[78]
*Bacillus subtilis* BJ–1	50	–	30	48	1.4 × 10^8^	74.4	8.58	[79]
*Bacillus subtilis* M–15	50	–	25	336	10^9^	96.5	–	[67]
*Candida tropicalis*	55	5.2	30	48	10^7^	88.6	15.24	[75]
*Candida tropicalis* ZD–3	50	–	30	48	10 g mycelia/Kg	94.6	10.76	[54]
*Candida tropicalis* ZAU–1	55	6.0	30	72	10^7^	92.29	–	[55]
*Saccharomyces cerevisiae* ZD–5	50	–	30	48	5 mL yeast	88.51	11.09	[54]
*Candida utilis*	50	–	30	24	5.0 × 10^5^	67.1	2.3	[80]
*Meyerozyma guilliermondii* WST–M1	45	–	30	72	3.0 × 10^8^	74.70	6.10	[71]
*Pichia pastoris* Y–2	50	–	30	48	20 g mycelia/Kg	58	–	[81]
*Saccharomyces cerevisiae*	50	–	28	48	60 mg yeast	25	–	[82]
*Aspergillus niger* ZD–8	50	–	30	48	10 g mycelia/Kg	85.15	22.23	[54]
*Pycnoporus sanguineus* CC400	60	–	28	360	–	98.95	–	[46]
*Geotrichum candidum* G07	62.19	–	30	48	10^7^	78.9	–	[83]
*Aspergillus niger*	50	–	28	48	10% (*v*/*w*)	–	8.42	[84]
*Pleurotus sajor-caju* and *Saccharomyces cerevisiae*	70	–	30	48	10^6^, 15% (*v*/*w*)	83.6	–	[85]
*Saccharomyces cerevisae* and *Aspergillus niger*	55	–	30	48	10^6^, 5% (*v*/*w*)	90.2	–	[86]
*Bacillus subtilis* ST-141 and *Saccharomycetes* N5	33.3	–	30	48	10^9^, 0.5% (*v*/*w*)	57.8	2.41	[87]
*Candida tropicalis* and *Saccharomycetes cerevisiae*	70	–	28	48	7.5% (*v*/*w*)	83.6	67.5	[88]
*Bacillus clausii* and *Saccharomyces cariocanus*	50	–	32	60	10^9^ and 5.0 × 10^9^	36.50	17.45	[89]
*Saccharomyces cerevisiae*, *Bacillus subtilis* and *Lactiplantibacillus plantarum*	54.5	5.72	30	168	10^5^, 1:1:1	89.14	8.02	[90]
*Candida tropicalis* and *Saccharomyces cerevisiae*	70	–	30	48	15% (*v*/*w*)	79.50	13.40	[91]

## Data Availability

No new data were created or analyzed in this study.
